# Expression of placental glycans and its role in regulating peripheral blood NK cells during preeclampsia: a perspective

**DOI:** 10.3389/fendo.2023.1087845

**Published:** 2023-05-03

**Authors:** Julio C. Bueno-Sánchez, Alejandra M. Gómez-Gutiérrez, Juan G. Maldonado-Estrada, Juan C. Quintana-Castillo

**Affiliations:** ^1^ Reproduction Group, Department of Physiology and Biochemistry, School of Medicine, Universidad de Antioquia, Medellín, Colombia; ^2^ Department of Obstetrics and Gynecology, School of Medicine, Universidad de Antioquia, Medellín, Colombia; ^3^ Red Iberoamericana de Alteraciones Vasculares en Trastornos del Embarazo (RIVATREM), Chillan, Chile; ^4^ One Health and Veterinary Innovative Research & Development (OHVRI) Research Group, Escuela de Medicina Veterinaria, Universidad de Antioquia, Medellín, Colombia; ^5^ Grupo Infettare, Facultad de Medicina, Universidad Cooperativa de Colombia, Medellín, Colombia

**Keywords:** glycosylation, preeclampsia, syncitiotrophoblast, NK cells activity, sialic acid (*N*-Acetyl neuraminic acid)

## Abstract

Preeclampsia is a pregnancy-related multisystem disorder characterized by altered trophoblast invasion, oxidative stress, exacerbation of systemic inflammatory response, and endothelial damage. The pathogenesis includes hypertension and mild-to-severe microangiopathy in the kidney, liver, placenta, and brain. The main mechanisms involved in its pathogenesis have been proposed to limit trophoblast invasion and increase the release of extracellular vesicles from the syncytiotrophoblast into the maternal circulation, exacerbating the systemic inflammatory response. The placenta expresses glycans as part of its development and maternal immune tolerance during gestation. The expression profile of glycans at the maternal–fetal interface may play a fundamental role in physiological pregnancy changes and disorders such as preeclampsia. It is unclear whether glycans and their lectin-like receptors are involved in the mechanisms of maternal–fetal recognition by immune cells during pregnancy homeostasis. The expression profile of glycans appears to be altered in hypertensive disorders of pregnancy, which could lead to alterations in the placental microenvironment and vascular endothelium in pregnancy conditions such as preeclampsia. Glycans with immunomodulatory properties at the maternal–fetal interface are altered in early-onset severe preeclampsia, implying that innate immune system components, such as NK cells, exacerbate the systemic inflammatory response observed in preeclampsia. In this article, we discuss the evidence for the role of glycans in gestational physiology and the perspective of glycobiology on the pathophysiology of hypertensive disorders in gestation.

## 1 Introduction

Preeclampsia is the most common hypertensive disorder in pregnancy, characterized by mild-to-severe microangiopathy of the placenta, kidney, liver, and brain (1–3). Before clinical signs of the disease appear, altered placental development exacerbates the local and systemic inflammatory status targeting the vascular endothelium (4–6). The systemic inflammatory response and clinical signs of preeclampsia end shortly after the fetus and placenta are removed during cesarean section, though hypertensive episodes may persist in some patients beyond the postpartum period. This situation increases the risk of renal disease or new episodes of preeclampsia in the subsequent gestation (7–9).

Preeclampsia is defined as a new onset of maternal hypertension after 20 weeks of gestation and systemic endothelial dysfunction manifested by new onset proteinuria (though not in all patients), hepatic dysfunction, and thrombocytopenia, among other symptoms (10, 11). Furthermore, preeclampsia exhibits diverse clinical manifestations, such as mild or severe, early or late onset (>34 weeks), or the presence or absence of intrauterine growth restriction (12–14). Pathological features of preeclampsia include shallow trophoblast invasion and poor spiral artery remodeling, resulting in placental hypoperfusion and intrauterine growth restriction (15, 16). These events occur during the first trimester of pregnancy and initially compromise the maternal–fetal interface, resulting in increased anti-angiogenic (17) and inflammatory factor production (18–22). Two pathophysiological stages have been proposed (23) to understand better the underlying mechanisms of preeclampsia: The first stage includes poor placentation, placental hypoperfusion, hypoxia, and trophoblast oxidative damage, followed by endothelial dysfunction and hypertensive clinical signs in the second stage (21, 24–26). Endothelial activation is intrinsic to the exacerbated systemic inflammatory response in severe preeclampsia (27), which involves NK cell activation rather than monocyte or lymphocyte activation (28, 29), resulting in coagulation dysfunction, insulin resistance, and hyperlipidemia (30).

In this context, the etiopathogenesis of preeclampsia is unknown, and the factors triggering its onset and progression to a multisystemic syndrome are unpredictable. Preeclampsia pathophysiology has traditionally focused on abnormal trophoblastic invasion, vascular inflammation, and the systemic inflammatory response (Croci et al., 2014). A more comprehensive approach is required to understand the occurrence of the multisystem syndrome (31). In this review, we focused on glycan recognition by peripheral NK cells and modulation of activation toward the cytotoxic phenotype to uncover new insights into molecular communication mechanisms between the trophoblast and the peripheral innate immune system. In the last two decades, a glycobiological perspective on pregnancy has emerged (32–35), and these new approaches have the potential to provide critical insights into the pathogenesis of preeclampsia and the development of severe clinical forms of the disease (36–43).

### 1.1 The role of glycans in cell-to-cell communication

The glycocalyx is the cell’s outermost layer of glycoconjugates, mainly glycoproteins and proteoglycans. The glycans bound to these glycoconjugates participate in ligand–receptor interactions in biological processes involving cell-to-cell interaction and function as a barrier and filter in endothelia (44, 45). In turn, the set of glycoprotein modifications complements the glycocalyx, and its more complex structure is encoded by nearly 10% of the transcribed genes that comprise the so-called glycome (46). The glycocalyx conformation may vary depending on the cell type, its activation and differentiation state, the cellular microenvironment, and the physiological or pathological conditions of the cells (47). Therefore, two of the systems in which the physiological role of glycocalyx has been studied are the immune system (reviewed in 48) and the vascular endothelium (reviewed in 49), particularly in pathological conditions (reviewed in 47, 50).

### 1.2 Structure of glycans

The glycocalyx is made up of monosaccharides or carbohydrates. In this review, glycan is preferred over carbohydrates because, unlike carbohydrates, not all glycans are monomers. Additionally, carbohydrate is often used interchangeably with components of intermediary metabolism. Glycans are composed of one carbonyl group, and the rest of the carbons contain hydroxyl groups. Carbonyl position and hydroxyl group orientation of atoms in asymmetric carbon determine monosaccharide structure. The anomeric carbon is derived from the carbonyl group; depending on the orientation of the anomeric carbon’s hydroxyl group, the monosaccharide exists in either a or b anomer (48). The identity, variety, and functions of glycans are determined by chemical modifications of the hydroxyl groups, such as oxidation, *N*-acetylation, and sulfation (51, 52). Finally, when glycans combine to form linear and branched glycan structures, the variety of glycans exponentially increases. The diversity of glycans is estimated to be 1.05 × 1012 of linear and branched glycan structures (53). The most common monosaccharides found in mammalian cells include glucose (Glc), galactose (Gal), mannose (Man), *N*-acetylneuraminic acid (NeuAc), *N*-acetylglucosamine (GlcNAc), *N*-acetylgalactosamine (GalNAc), glucuronic acid (GlaA), xylose (Xyl), and fucose (Fuc) (48).

The DNA code does not determine protein modifications by enzymatic glycosylation because cellular enzymatic expression varies between species, cellular microenvironments, and cell types (54). Besides, glycosylation is a remarkably well-conserved enzymatic process (55). Specific changes in each glycan sequence will depend on the expression of glycosidases and glycosyltransferases, the availability of nucleotide sugar donor sources, and external influences mediated by cytokines and hormones in the cell microenvironment (56, 57). Glycosidases are enzymes that catalyze the hydrolysis of a bond linking a sugar of a glycoside to alcohol or another radical in the glycan molecule, whereas glycosyl transferases are enzymes that catalyze the transfer of glycosyl groups in biochemical reactions (58).

**Figure 1:**
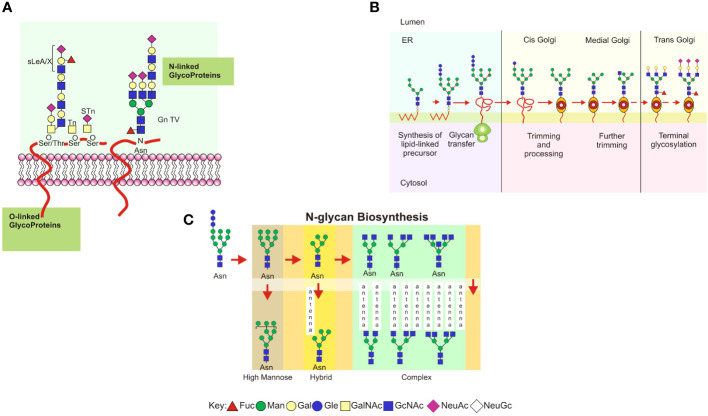
**(A)** Schematic representation of the main types of glycoproteins expressed in mammalian glycocalyx. Types of cell-surface glycans, including *N*-linked and *O*-linked glycans from glycoproteins. Each glycan has a common core whose terminal or internal sequences can vary, leading to a complex diversity among cells or defined microenvironments. **(B)** A consensus sequence for *N*-glycosylation (Asp-X-Ser/Thr, designated sequon) is used as an acceptor in polypeptide chains of a preformed oligosaccharide attached to a dolichol phosphate in the endoplasmic reticulum. The oligosaccharide glucose_3_–mannose_9_–*N*–acetylglucosamine_2_ is transferred to the protein chain by an oligosaccharyltransferase acting as a chaperone and modulating its folding to transport toward the Golgi complex. Trimming and processing of the attached glycan are performed in Golgi compartments by specific glycosyltransferases and glycosidases for each nucleotide sugar donor. It is in the Golgi apparatus that glycans become oligomeric and branched. **(C)** Eukaryotic *N*-glycan biosynthesis in the endoplasmic reticulum and Golgi complex. Processing intermediates of *N*-glycans preserve a unique sequence core Manα1-6 (Manα1-3) Manβ1-4GlcNAcβ1-4GlcNAcβ1-Asn-X-Ser/Thr. Three types of N-glycans are found: oligomannose, complex, and hybrid.

The structural diversity of the mammalian glycome is supported by the synthesis of glycans and their enzymatic regulation, which occurs in the endoplasmic reticulum and Golgi apparatus when added to proteins. If the glycan binds to the nitrogen of asparagine in the protein, it is called *N*-glycosylation or *N*-glycan; if it binds to the oxygen of a serine or threonine, it is called *O*-glycosylation or *O*-glycan (**Figure 1A**). In the case of proteoglycans, an unbranched polysaccharide chain composed of disaccharide units of a sulfated amino sugar (*N*-acetylglucosamine or *N*-acetylgalactosamine) and uronic acid (iduronic or glucuronic) is attached to a large protein core in a serine residue (59–61).

### 1.3 *N*-linked glycosylation

In *N*-glycosylation, a series of glycosyltransferases use nucleotide-activated sugar donors as substrates to form the mannose oligosaccharide 5-*N*-acetylglucosamine (**Figure 1A**). Oligosaccharide portions formed in the endoplasmic reticulum, specifically terminal glucose, and mannose, combined with molecular chaperones and thiol-disulfide oxidoreductases, help to fold newly synthesized proteins (62). The *N*-glycosylated polypeptide is delivered into the Golgi apparatus following interaction with cargo receptors in the vesicular transport system (63). If the protein is misfolded, it is translocated to the cytosol, deglycosylated by the enzyme peptide *N*-glycanase, or degraded by the ubiquitin/proteasome. In this pathway, glycosidases and mannosidases in the endoplasmic reticulum trim the *N*-glycans bound to the nascent protein, resulting in intermediate processing forms (**Figure 1B**; 64). The mannose residues are trimmed in the endoplasmic reticulum and subsequently in the Golgi complex, where the processed glycans undergo the addition of *N*-acetylglucosamine (*N*-acetylglucosaminylation), galactose (galactosylation), sialic acid (sialylation), or sulfate (sulfation). The intermediate forms of *N*-glycans share a common glycan core consisting of Manα1-6 (Manα1-3) Manβ1-4GlcNAcβ1-4GlcNAcβ1-Asn-X-Ser/Thr and have been classified into three types: (a) oligomannose, in which the mannose residues are attached to the core; (b) complex, in which the mannose residues are trimmed and replaced by *N*-acetylglucosamine, resulting in new elongated branches attached to the *N*-acetylglucosamine residues called “antennae”; and (c) a hybrid, in which only the mannose residues attached to Manα1-6 are conserved, while the mannose attached to Manα1-3 is trimmed and replaced in an antennae sequence by a branched *N*-acetylglucosamine (**Figure 1C**; reviewed in 64, 65).

### 1.4 *O*-linked glycosylation

*O*-linked glycosylation involves the formation of *O*-glycosidic bonds between oligosaccharide chains *via N*-acetylgalactosamine and GalNAc (1β-3), which is found in mucin glycoproteins (**Figure 1A**) (66). These glycans are mucins with GalNAc *O*-linked to Ser or Thr. *O*-glycosylation occurs in mucin domains containing many serine, threonine, and proline sequences. *O*-glycan processing occurs in the Golgi apparatus and has little impact on the early stages of protein folding. In mammalian mucins, the first attached GalNAc can be replaced by GlcNAc, GalNAc (linkage 1α-3), or Gal, forming up to eight different core structures: core 1: Galβ1-3GalNAc; core 2: GlcNAcβ1-6(Galb1-3)GalNAc; core 3: GlcNAcβ1-3GalNAc; core 4: GlcNAcβ1-6(GlcNAcβ1-3)GalNAca; core 5: GalNAcα1-3GalNAc; core 6: GlcNAcβ1-6GalNAc; core 7: GalNAcα1-6GalNAc; and core 8: Galα1-3GalNAc (67). The most common core sequences are 1 and 2; cores 3 and 4 are only found in mucins. The core structures can be substituted or elongated with sialic acid residues. *O*-glycan biosynthesis is initiated by the enzyme *O*-GalNAc transferase, which transfers the GalNAc residue of UDP-GalNAc to serine or threonine residues in a protein with the α-configuration (68). *O*-glycan processing does not require dolichol derivatives or specific glycosidases, and the mRNA levels of glycosyltransferases are critical factors for their assembly (69). Additionally, glycosyltransferases may form complexes with other proteins, influencing their activities, or metal ion concentrations in the Golgi apparatus may regulate them. The presence of an unsubstituted GalNAc on *O*-glycoproteins forms the Tn antigen in tumor metastasis (70), and GalNAC can be directly substituted with sialic acid to form the sialyl-Tn (STn) antigen. These glycans are truncated and not used in subsequent elongation reactions (68, 70–72).

### 1.5 Glycan receptors and ligand-receptor interactions

Glycan receptors are proteins with carbohydrate recognition domains (CRDs) called lectins. Lectins bind to clustered CRDs with high affinity *via* (a) binding to multiple epitopes on a single oligosaccharide or polysaccharide, (b) multiple glycans attached to a single protein scaffold, and (c) binding to adjacent glycoproteins or glycolipids in the cell membrane. Plants produce the well-known lectins, followed by those isolated from animals and pathogenic microorganisms (73). The various types of CRDs are classified into four major lectin groups: (a) Siglecs (short for sialic acid binding immunoglobulin-type lectins) or I-type lectins, in which the CRDs are formed by an immunoglobulin domain fold (sialic acid is the major glycocalyx component related to immune system regulation); (b) galectins (predominantly binding to *β*-galactosidase), which contain CRDs formed from a beta-like fold of the protein; (c) C-type lectins, in which the sugars bind directly to a calcium ion attached to the CRD; and (d) lectins containing R-type CRDs, whose structure is related to the plant toxin ricin (reviewed in 74). The most important biological functions of ligand–receptor interactions between glycan ligands and lectin-like receptors include cell adhesion, intracellular trafficking, glycoprotein elimination and turnover, cell signaling, and pathogen recognition (48, 65, 74).

### 1.6 Trophoblast differentiation in human placental development, hypoxia, and glycan expression

**Figure 2:**
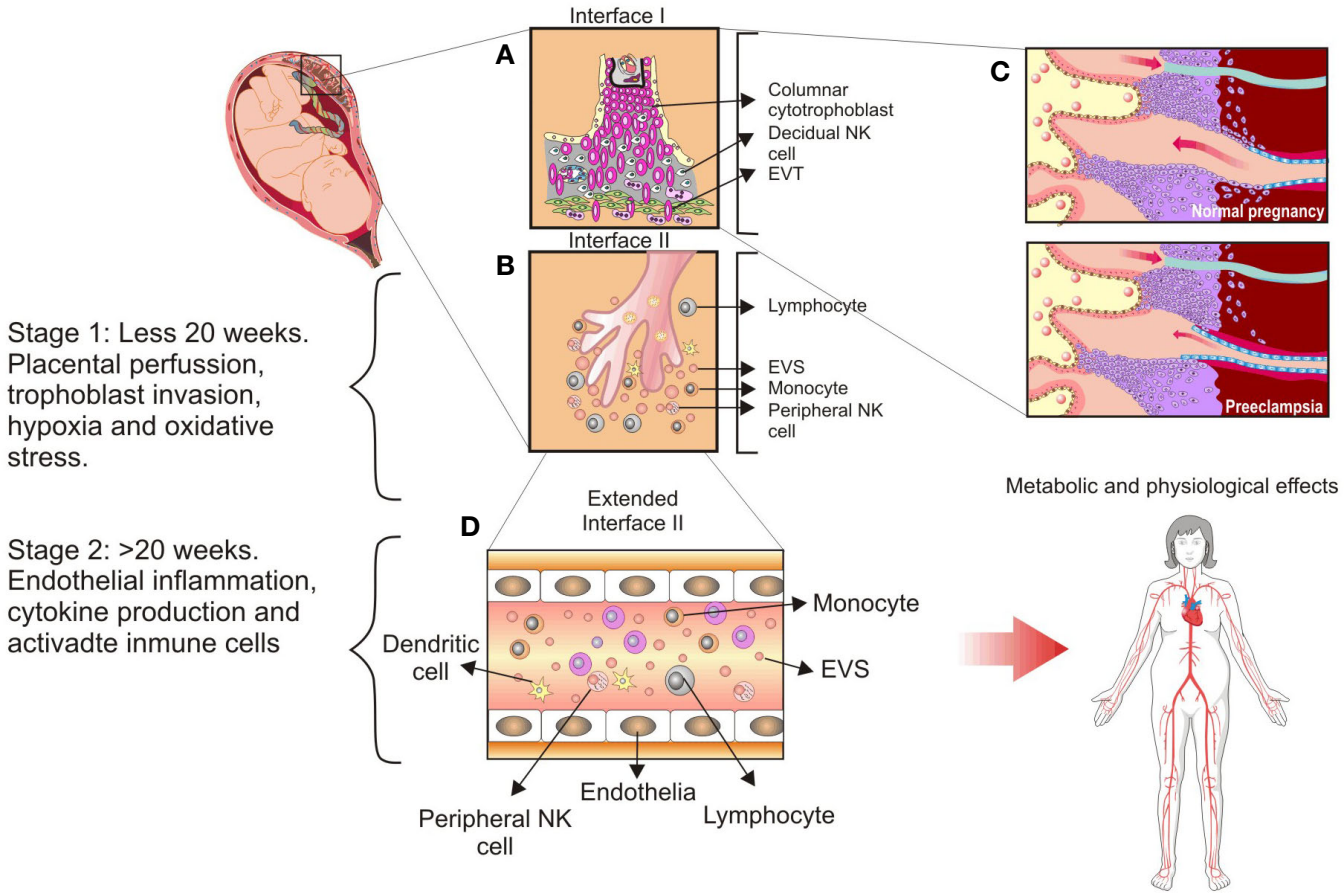
Main cellular components found at the maternal–fetal interface and the proposed stages of preeclampsia. **(A)**, Interface I: Villous tree represents a tertiary villous and its predominant cellular components at interface: Columnar cytotrophoblast, anchoring trophoblast, and extravillous trophoblast subsets. **(B)**, Interface II: Floating villi are in contact with maternal blood flow and release syncytiotrophoblast extracellular vesicles into circulation. Peripheral Natural Killer (NK) cells are highlighted as a significant maternal innate immune system component. **(C)**, Spiral arteries remodeling in the placental bed occurs extensively in a normal pregnancy but not in preeclampsia. **(D)**, Extended interface II: Syncytiotrophoblast extracellular vesicles are delivered into maternal circulation and promote the systemic inflammatory response observed during normal pregnancy. This inflammatory response is exacerbated in the case of women with preeclampsia, and it is linked to abnormal placental invasion, low placental perfusion, and increased oxidative stress accompanied by chronic hypoxia. CC, columnar cytotrophoblast; DC, dendritic cells; EVs, syncytiotrophoblast extracellular vesicles; EVT, extravillous trophoblast. This figure is adapted from Sargent et al. (232).

The human placenta is an autonomous and transient anatomical structure that allows the mother and fetus to exchange nutritional, gas, and waste products. The trophoblast layer mediates fetal growth and maternal pregnancy adaptation generating a new vascular bed in the placental interface. These trophoblast cells are the first to separate from the developing blastocyst, giving rise to cytotrophoblast stem cells (75, 76). Placental villous tissue develops from primary to secondary and tertiary villous tissue with the progressive development of villous tree circulation complexity. Primary villi containing cytotrophoblast and syncytiotrophoblast cells predominate during the first four weeks of gestation. Secondary villi develop in the fifth week of gestation, with the extraembryonic mesoderm forming villi and covering the surface of the chorionic sac to provide a framework for intra-villi blood circulation. In the third stage of chorionic villi development, which occurs during the sixth week of gestation, mesenchyme differentiates into blood vessels and cells, resulting in the arteriocapillary framework that fuses with placental vessels into the connecting stalk (reviewed in 77). Besides, they develop with greater complexity, resulting in the formation of (a) stem villi or anchoring villi, from which cytotrophoblast cells migrate into the decidua and become extravillous cytotrophoblast (**Figure 2A**); (b) branched villi, which grow from the stem villi and represent the most critical portion for exchange with maternal blood from the placental bed through the intervillous spaces (**Figure 2B**); (c) terminal villi, which consist of protrusions caused by trophoblast proliferation due to the coiling of the fetal capillaries within the mature intermediate villi beginning in the third trimester of gestation; and (d) chorionic plate, which is the region at the base of the villi through which the placental arteries and vein pass (**Figure 2A**; 78–80). The villous cytotrophoblast stem cell population supports the differentiation of trophoblast into two major cell lineages, syncytiotrophoblast and the invasive trophoblast (named extravillous trophoblast [EVT]; 76, 81–83). The most invasive trophoblast wave occurs when EVT cells infiltrate the endothelium of maternal spiral arteries to come into direct contact with systemic circulation, forming placental villi and a maternal–fetal interface (78). The cell layer responsible for maternal–fetal nutrient and gas exchange is the well-differentiated syncytiotrophoblast cells, which cover the anchoring, branched, and terminal villi (84–86).

**Figure 3:**
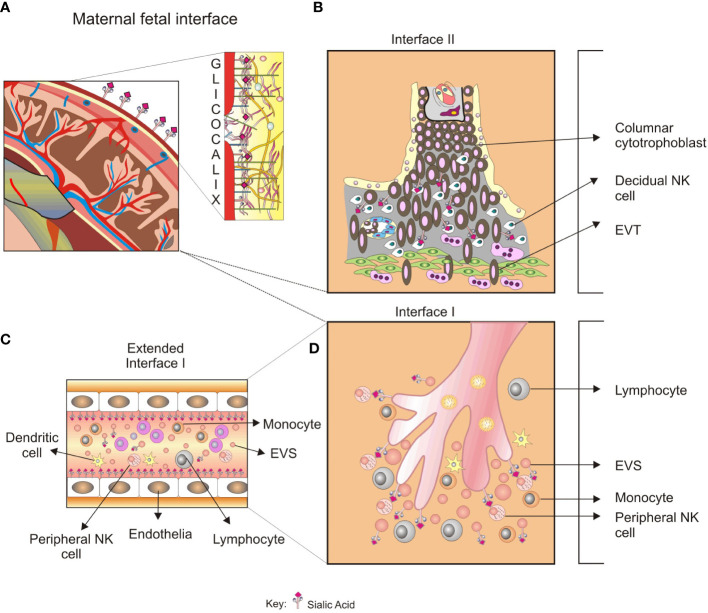
A glycobiological view of the systemic inflammatory response in preeclampsia. **(A)**, Sialylated glycoproteins and glycoproteins with LacNAc glycotopes, among others glycoconjugates in the trophoblast glycocalyx, are expressed in maternal–fetal interface II. **(B)**, The recognition by LacNAc by Gal-1 promotes angiogenesis in the placental bed, and impaired balance in the Gal-1 function results in a PE-like syndrome. Furthermore, defective trophoblast invasion has been associated with specific blocking of Gal-1. **(C)**, Glycoconjugates could be recognized by peripheral NK cells in the maternal–fetal interface I, modulating the NK cell activity. The released syncytiotrophoblast EVs found in preeclampsia could transport glycoproteins with immunomodulatory activity and increase the systemic inflammatory response. **(D)**, EVs released from maternal–fetal interface II spread into the systemic circulation and promote endothelial activation. The type and quantity of VES found in preeclampsia could initiate endothelial damage and exacerbate inflammation. The role of glycan recognition in syncytiotrophoblast VES-mediated endothelial damage is not elucidated yet.

One of the most intriguing aspects of placental development during the first trimester is that the partial occlusion of the maternal spiral artery lumen by the extravillous invading endothelial trophoblast (see 87) results in a temporary condition of placental hypoxia, with approximately 20-mm Hg O2 concentration during the first trimester compared with 60 and 40-mm Hg during the second and third trimesters, respectively (**Figure 2C**) (88). During the first trimester of gestation, hypoxia at the maternal–fetal interface shapes placental development, which involves cellular and molecular adaptations to compensate for the low oxygen tension. As a result, trophoblast cells are a highly dynamic cellular type responsible for proper placental development. However, these processes are disrupted during pathological gestation conditions, resulting in a loss of placental architecture and abnormalities in fetal growth and development (**Figure 2C**) (89).

Extravillous trophoblast cells exhibit an invasive phenotype that shares molecular mechanisms with tumor cells, such as binding to the extracellular matrix, extracellular matrix degradation by metalloproteases, and migration through the extracellular matrix mediated by *N*-linked complex-type glycans (90–92). Truncated forms of *O*-glycans, such as core 1 (the Thomsen–Friedenreich [T or TF] antigen) and core 1 with a sialic acid substitution at the α2-3 bond to Gal and at the α2-3 bond to GalNAc to form the sialyl-T antigens, are associated with metastasis in some tumors (93, 94). Furthermore, the environment of tumor cells and EVTs are hypoxic, which confers invasive characteristics. About 60% of solid tumors have an oxygen partial pressure of less than 10 mm Hg compared with 50- to 60-mm Hg observed in adjacent non-tumor tissues (95). These values are comparable with the hypoxic state of the maternal–fetal interface during the first trimester of gestation (96–99). Hung and Burton (100) and Robins et al. (99) identified fluctuations in placental oxygen tension as a determinant of trophoblast invasion and differentiation associated with maternal spiral artery endothelial remodeling and placental angiogenesis.

There is low intrauterine oxygen tension at early implantation, and these oxygen levels are maintained during the first trimester of pregnancy. However, when the placental vascular bed is established, the degree of differentiation of trophoblasts to EVTs and interstitial and endovascular EVT subsets between 8 and 24 weeks of gestation promote increased oxygen tension (97, 99, 101, 102). Besides, the microenvironment into which the EVT invades the decidual stroma influences the release of soluble factors (e.g., immunomodulatory, pro-inflammatory, and anti-inflammatory cytokines, growth, and angiogenic factors), and oxygen concentration influences changes in the invasion process. As previously mentioned, physiologically hypoxic conditions (2%–3% compared with the usual 20% oxygen) in the first trimester promote differentiation toward an invasive pathway and establish a new placental vascular bed (96, 103).

Kang et al. (21) proposed that a failure of trophoblast invasion causes preeclampsia, and Goldman, Wohl, and Yagel (24) proposed that preeclampsia is caused by complex pathological events related to shallow invasion of endovascular and interstitial trophoblast, which reduces the transformation of spiral arteries into low-capacitance vessels (**Figure 2C**). Despite all evidence, it is inconclusive that the diseases have a distinct placental etiology. Thus, Staff (26) revised the concepts of the two-stage placental model of preeclampsia, which proposed a stage 1 caused by placental dysfunction and a stage 2 related to maternal clinical syndrome (25). In stage 1, maternal factors include a lack of tolerance to allogeneic trophoblasts, impaired placentation, altered spiral artery remodeling, and an oxidative stress state of the placenta. In stage 2, the impaired placenta and trophoblast stress send stress signals to the maternal endothelium, resulting in widespread vascular inflammation and the clinical signs of early-onset and late-onset preeclampsia (26). These concepts have recently been validated by evidence of atherosclerosis and spiral artery remodeling failure as determinants of preeclampsia and other pathological gestation conditions. Furthermore, Staff et al. (104) reaffirmed their proposal for a multistage placental model of preeclampsia.

### 1.7 HIF and hypoxia-related signaling

Low oxygen tension at the maternal–fetal interface promotes the accumulation of hypoxia-inducible factor, HIF-1, a regulator of oxygen homeostasis (105). HIF-1 is a heterodimeric protein composed of a constitutively expressed *β* subunit and an *α* subunit regulated by the partial oxygen pressure (pO2; 106). Low oxygen concentrations promote cell invasion and progressions by activating more than 60 putative genes after HIF-1 receptor activation (HIF-R) (107–109). Human cytotrophoblast cells express HIF-1α (20, 110), which regulates the expression of target genes in cells subjected to hypoxia, including transferrin receptor (TfR1), vascular endothelial growth factor (VEGF), erythropoietin, endothelin-1, leptin, and glucose transporter 1 (106). HIF-1α regulates the iron and 2-oxoglutarate-dependent dioxygenase family enzymes known as prolyl hydroxylases (PHD1, PHD2, and PHD3) and asparaginyl hydroxylases (95). The PHD2 enzyme is responsible for hydroxylating one of the two proline residues present in HIF-1α (pro402 and pro564), promoting its interaction with the von Hippel-Lindau protein (pVHL) and subsequent polyubiquitination and degradation in the proteasome (103). When a cell is deprived of its oxygen supply, hydroxylation, and subsequent degradation of HIF-1α are significantly reduced, resulting in accumulation (111). HIF-1α accumulates in the nucleus, where it forms a complex with HIF-1β (HIF-1α/HIF-1β heterodimer) that is selectively expressed in the placenta (112, 113). The HIF-1α/HIF-1β heterodimer binds to hypoxia-responsive elements, acting as transcription factors for genes involved in cellular stress response (114). Thus, hypoxia stimulates tumor cell and trophoblast migration and invasion, and the invasive phenotype is activated *via* multiple mechanisms, including direct and indirect regulation of the epithelial to mesenchymal transition and upregulation of proteolytic enzymes, primarily matrix metalloproteinases (115, 116).

During the first trimester of gestation, hypoxic conditions stimulate the production of soluble factors associated with placental angiogenesis and immune tolerance to trophoblast invasion at the maternal–fetal interface (117). However, hypoxia in the second or third trimester may promote an increase in placental apoptosis and a significant decrease in trophoblast invasion, exacerbating the pro-inflammatory state at the maternal–fetal interfaces (99, 101, 118). The significant reduction in maternal spiral artery remodeling during the trophoblastic invasion and the consequent decrease in placental perfusion could correlate with overexpression of HIF-1α, VEGF, soluble FMS-like tyrosine kinase (sFlt1), and soluble VEGF receptor (sVEGFR-1), all elicited by HIF (119, 120). Failure to detect low oxygen tension has been proposed as a cause of early-onset preeclampsia (121), supporting the concept of abnormal placental development as one of the triggering processes (122).

### 1.8 Glycans as mediators between the placenta and peripheral circulation

The glycosylation profile of trophoblast and glycans do change during endometrium trophoblast invasion. Because of their molecular versatility, they form new affinities and cell–to-cell crossovers and develop complex intercellular structures supporting trophoblast differentiation (123). Changes in glycan profiles could be new biomarkers for diseases such as preeclampsia (124, 125). These changes involve the underexpression and overexpression of naturally occurring glycans in response to the environment- or stress-induced signaling (126). Thus, glycosylation of glycoproteins may contain previously unknown patterns that control stem cell differentiation, phenotype changes, invasion, and mobilization (60, 127), vasculogenesis (128, 129), and extravillous trophoblast invasion (130). The maternal immune system regulates trophoblast differentiation and invasion at multiple levels: locally in the maternal–fetal interface I by uterine NK-to-dendritic cell interaction and systemically in the maternal–fetal interface II by trophoblast-derived extracellular vesicles (EVs) (**Figure 2D**) or in the vascular compartment by VEGF production (**Figures 2A–D**; 131, 132). In this regard, Clark et al. (133) proposed that the human embryo–fetus’ primary defense mechanism for protecting itself from maternal immune system recognition and rejection is the induction of specific immunosuppressive oligosaccharides in glycoconjugates of placental tissues (133). The syncytiotrophoblast in the full-term placenta expresses up to 95% *N*-linked oligosaccharides with complex sugars, with the remaining 5% being high mannose type (134). These glycan structures were immunosuppressive because the oligosaccharide membrane fraction isolated from syncytiotrophoblast decreased 3H thymidine uptake in a mixed leucocyte reaction (135).

Glycosylation changes can manifest in various ways, including impairing naturally occurring glycans through the influence of environmental or stress-induced signaling pathways. These changes can lead to either under-expression or overexpression of glycans. Additionally, the expression of glycans that are typically restricted to embryonic tissues can occur during the development of a tumor phenotype. (94). Changes in glycosyltransferase levels can lead to modifications in the core structure of N-linked and O-linked glycans, e. g. the oligosaccharide size and branching of N-linked glycans. The increased activity of N-acetylglucosaminyltransferase V (GlcNAc-TV, or MGAT5; the enzyme that leads to β1,6GlcNAc branching) promotes β1-6- linked branching and the invasive phenotype in transfected non-metastatic clones of murine mammary carcinoma (136). The placentation process resembles cancer metastasis in the invasion waves during the first trimester. Therefore, the expression of β1-6- linked branching increases in the trophoblast layer when it is detected by phytohemagglutinin lectin blotting (137). The increased branching creates additional sites for terminal sialic acid residues, which, in conjunction with the corresponding upregulation of sialyltransferases, ultimately leads to an increase in global sialylation. *N*-glycan structures obtained from syncytiotrophoblast proteins are highly sialylated during pregnancy, as was confirmed by mass spectrometry (43), which could explain syncytiotrophoblast resistance to NK cell—and other cytolytic leukocytes—mediated cytolysis (138). These patterns of glycosylation were confirmed later by Chen et al. (43), who proposed that biantennary bisecting type *N*-glycans(a particular type of glycan modification consisting of four-linked β1 GlcNAc structure attached to the core β-mannose residue expressed in syncytiotrophoblast), could protect class I MHC non-expressing trophoblast cells from NK cell-mediated cytotoxicity. However, further experimental evidence is needed to confirm this. A knockdown of the MGAT5 enzyme increases the migration capacities of first-trimester human placental villi, which are correlated with the upregulation of metalloproteases (MMP-9) and the downregulation of the invasion inhibitor TIMP1/2 (139). Furthermore, the ischemia-reperfusion insult of the placenta may promote the shallow trophoblastic invasion observed in preeclamptic pregnancies, whereas specific changes in *N-*glycans could be involved in migration and differentiation processes. Thus, N-acetylglucosaminyltransferase III (GnT-III) catalyzes the bisecting GlcNAcβ1 modification, which can suppress the processing and branching of the glycan catalyzed by GnT-V (140).

Reduction of biantennary de-sialylated non-fucosylated *N*-glycans was reported in first-trimester decidual tissue as a normal finding (141), but no reports on the expression by placental villi tissue were found. Furthermore, Whyte and Loke (142) reported increased sialylation of trophoblast glycoproteins compared with fetal and tumor cells. According to Jeschke et al. (143), one possible mechanism of immunosuppression in human gestation is the expression of TF antigen at sites of trophoblast invasion.

Placental proteins have different glycosylation patterns depending on gestational age or extracellular cell microenvironmental changes. For instance, Dell et al. (56) reported that glycodelin-A —a potent immunosuppressive protein in the human placenta— expresses biantennary *N*-glycans with the bisecting GlcNAc sequence and sialylated complex *N*-glycans (143–145). The syncytiotrophoblast layer of chorionic villi produces human chorionic gonadotrophin (hCG) throughout human pregnancy (146). hCG, an *N*-glycan glycoprotein, is also secreted by EVT during trophoblast invasion in a hyperglycosylated form, exerting potent immunomodulatory effects (147, reviewed in 146).

Changes in glycosylation profiles of placental glycoproteins associated with preeclampsia have been proposed to be related to oxidative stress-promoting hypoxia in the placental bed and the consequent accumulation of the HIF heterodimer, as mentioned above. For example, Lewis*Y*, a well-recognized glycan associated with angiogenesis, was found in cytotrophoblast cells and villous trophoblastic cells in cases of severe preeclampsia (148) and unexplained miscarriage (149). These results could be explained by a compensatory mechanism triggered by an increase in angiogenic factors, common in pregnancy-related hypertensive disorders, such as preeclampsia (28, 150). Besides, changes in glycan expression at the maternal–fetal interface caused by chronic hypoxia could be associated with increased lectin expression in preeclampsia. This is the case with galectins (Gal), soluble non-glycosylated proteins with a unique sequence motif in their CRDs that show an increased affinity for *N*-acetylated disaccharides such as *N*-acetyl-galactosamine (LacNAc; Galβ1,4GlcNAc) and similar structures. In murine models, Gal-1 exhibits proangiogenic functions during the early stages of pregnancy, promoting decidual vascular expansion *via* VEGF receptor 2 (VEGR2) signaling. A specific blockage of Gal-1 promotes defective trophoblast invasion and impaired maternal spiral artery remodeling, resulting in a PE-like syndrome (151). Gal-1 expression was significantly upregulated in the decidua of preeclamptic placentas and the villous trophoblast of placentas in women with hemolysis, elevated liver enzymes, and low platelet (HELLP), which is consistent with previous findings (144).

### 1.9 Changes in glycosylation are mediated by hypoxia and inflammation

As mentioned above, hypoxia affects metabolism cells and glycosyltransferase expression in placenta and tumors (139, 140, 152) such as colon cancer cell lines (153, 154) and the immortalized prostate cell line RWPE1 (155). It also promotes the transcription of genes encoding fucosyltransferases and sialyltransferases. Although the role of HIF-1α in placental glycosylation remains unknown, indirect evidence in tumor tissues indicates that HIF-1α upregulation increased fucosyltransferases (FUT)-1-2 mRNA levels and overexpression of fucosylated proteins on the surface of pancreatic adenocarcinoma cells (156). Hypoxia appears to alter endothelial cell glycosylation, inducing the expression of *N*-glycoproteins containing glycans with less α2,6-linked sialic acid, elongated poly-LacNAc (Gal-GlcNAc) residues, and β1,6-linked *N*-glycan structures branching (157, 158). Furthermore, pathophysiological changes of the endothelial surface layer occur following a variety of insults, among these, hypoxia associated with ischemia/reperfusion injury (159). The oxidative and inflammatory stress accompanying hypoxic insult promotes glycocalyx damage *via* reactive oxygen species (Ros), increasing vascular permeability and perivascular inflammation. These ROs-mediated changes to the glycocalyx are dependent on Ca2+ signaling and possibly matrix metalloproteinase activation (160). However, another signaling pathway could be implied in the hypoxic insult associated with glycosyltransferase expression.

Additionally, HIF-1 activates tumor and placental inflammatory signaling, particularly nuclear factor-kappa B (NF-κB) transcription. Inhibitory IκB dissociates from NF-κB during inflammation, allowing for nuclear translocation and multiple gene transcriptions, mainly IL-6, cycloxygenase-2, and matrix metalloproteinase 9 (161, 162). These findings imply that HIF-1 and NF-κB interaction activates the transcription factors of these genes. In preeclampsia, NFκB expression may stimulate pro-inflammatory and anti-angiogenic protein production, promoting oxidative stress, inflammation, and vascular dysfunction. On the contrary, in normal pregnancies, NFκB promotes placental cellular migration, invasion, and angiogenic protein production (163). In preeclamptic women, local placental inflammation and oxidative stress are associated with placental hyperactivation of NfκB and its release into the maternal circulation, with levels nearly 10-fold higher than in normal pregnancies (163, 164).

Abnormal placental blood perfusion increases hypoxia-induced placental-derived circulating agents and sFlt1 production (120). HIF-1α, the primary regulator of oxygen hemostasis in the placenta, induces TGF-β production (165, 166). The β3 isoform of TGF-β suppresses trophoblast trans-differentiation, reducing its differentiation toward EVT and invasive capacity (165). Therefore, reduced growth of maternal–fetal interfaces 1 and 2 could restrict blood perfusion and promote EV release into the general circulation, contributing to the exacerbated pro-inflammatory status observed after 20 weeks of gestation (167). Placental hypoxia occurs in diseases such as gestational iron deficiency anemia, associated with increased accumulation of HIF-1α, similar to preeclampsia. Additionally, alterations in iron status have been associated with hypertensive disorders of pregnancy (168–171). Glycosylation profiles induced by hypoxia and placental inflammation have been proposed to alter the exchange of macronutrients and micronutrients across the syncytiotrophoblast (172, 173). Iron is transferred from the mother to the fetus *via* the placenta after being taken up by TfR1 at the apical membrane of the syncytiotrophoblast (174–177). TfR1 contains three *N*-glycan and O-glycan chains in its extracellular domain (178–180). TfR1 expression may be altered in hypoxic conditions, such as in women with diabetes, due to post-translational modifications, including glycosylation (181). Thus, women with preeclampsia whose placenta is exposed to prolonged hypoxia may have elevated levels of TfR1, one of the HIF-1 target genes. In preeclamptic women, the galactose-*N*-acetylglucosamine and terminal mannose patterns detected by DSA and GNA lectins, respectively, were overexpressed in TfR1. In addition, TfR1 expressed α2-3 sialic acid rather than α2-6 sialic acid, and preeclamptic placentas overexpressed α2-3 sialic acid (182). Depleting α2-6 sialic acid may promote increased binding of gal-3 to terminal galactose in TfR1 and formation of the transferrin–iron–TfR1 complex during iron uptake (183, 184). Furthermore, overexpression of α2-3 sialic acid in TfR1 of preeclamptic placentas may be associated with resistance to cell membrane scission, which is consistent with the findings of Rutledce and Enns (185), where removal of α2-3 sialic acid released TfR1 protein into the culture medium. We wonder if differences in TfR1 glycosylation patterns in preeclamptic women have implications for TfR1 exportation to the cell membrane or ligand affinity modifications, events that could affect iron uptake by the placenta resulting in impaired fetal nutritional status and, in some cases, intrauterine growth restriction.

### 1.10 Role of glycan recognition by cells of innate immunity in the pathogenesis of the exacerbated systemic inflammatory response in preeclampsia

Because preeclampsia is a preclinical condition characterized by a prolonged hypoxic state, it may be associated with an increased release of EVs and soluble factors capable of initiating a systemic inflammatory response and impairing endothelial function (131). Murrieta-Coxca et al. (186) recently proposed that EVs could play a critical regulator of feto-maternal tolerance by transferring allogeneic material from fetal to maternal immune cells to remodel their function (in the context of fetal allograft tolerance) and fetal–maternal microchimerism. Darmochwal-Kolarz et al. (187) reported that in preeclamptic patients, there was an imbalance of pro-inflammatory cytokines (IL-2 and IFN-γ) compared with the anti-inflammatory profile (IL-4 and IL-10) characteristic of mitogen-activated CD4+/CD8+ T lymphocytes and NK cells. However, studies on cytokine production by NK cells in preeclampsia used chemical stimulation with Phorbol Myristate Acetate (PMA) and Ca2+ ionophores, which could alter the cytokine profile observed in these assays (188).

The pro-inflammatory profile of NK cells could be induced by lectin-mediated glycan recognition at maternal–fetal interface 2, where peripheral immune cells come into contact with the syncytiotrophoblast layer in circulating microvilli. Under hypoxic conditions, for example, the glycan profile of invasive tumor cells and tumors may change, revealing truncated glycans such as Tn antigen or polysialylated *N*-glycan complexes (93, 189, 190). Recognition of these glycans by NK cells is mediated mainly by lectin-like and immunoglobulin-like receptors (called Siglecs; 191). On the other hand, the functional profile of peripheral NK cells is known to depend on their CD56 phenotype. Therefore, the CD56bright NK cell subset can promote the PMA and the Ca2+ ionophore-induced IFN-γ and TNF-α intracellular production (192). In non-pregnant women, the major NK cell subset is IFN-γ or TNF-α producing NK cells (NK1 profile), and IL-4, IL-5, and IL-13 producing NK cells (NK2 profile) or IL-10/TGF-β non-producing PMA-stimulated CD56bright and CD56dim NK cells (193–195). In pregnant women, the cytokine profile observed in NK cells changes to IL-10-producing NK cells; IFN-γ, TNF-α, IL-4, IL-5, or TGF β-producing CD56dim and CD56bright subsets corresponding to NK regulatory and NK3 profiles, respectively, with no difference between these subsets (194). This way, we evaluated the activation of peripheral NK cells before the more advanced stages of preeclampsia in women with early-onset severe preeclampsia without HELLP syndrome. Monocytes, T lymphocytes, NKT cells, and NK cell proportions were not significantly different compared with healthy pregnant women. Moreover, CD56dim and CD56bright NK cell subsets from the early-onset of severely preeclamptic women significantly produced more intracellular cytokines than NK cells from healthy pregnant women, with no cytokine imbalance. However, peripheral NK cells demonstrated increased cytotoxic activity *in vitro* (28), consistent with reports of increased NK cell cytotoxic activity in NK cells from mild preeclampsia cases (196). Besides, an increased percentage of NK cells bearing the CD96-NKGA/C receptors and CD56bright–NKG2C and NKG2A positive in NKdim and NKbright subsets (28). CD96-NKG2 heterodimers are lectin-like receptors that recognize protein-bound terminal glycans. They are members of the C-type lectin-like receptor superfamily, which includes six NKG2 molecular species (A, B, C, D, E, and H).

The heterodimer CD94/NKG2A is an inhibitory receptor with intracellular motifs that negatively regulates activation signaling for cytotoxic activity and anti-inflammatory cytokine production (197). Both CD94/NKG2 heterodimers can bind to non-classical HLA molecule HLA-E; however, their affinity varies: CD94/NKG2A binds to HLA-E with low affinity and very rapid association and dissociation rates, whereas CD94/NKG2C binds to HLA-E with nearly 10-fold lower affinity than CD94/NKG2A (198). This affinity is determined by the peptide sequence bound to HLA-E. However, evidence suggests a direct recognition of NKG2A/C receptors mediated by α2-3 sialic acid, a terminal residue of sialic acid monosaccharide-containing glycoproteins (199). NKG2A and NKG2C receptor expression on peripheral blood NK cells was reduced in women with preeclampsia (200). EA.hy296 endothelial cell line expresses HLA-E, and serum from severely preeclamptic women increased its expression (201). In severe preeclampsia, NK cell adhesion to the endothelium is significantly higher than in normal pregnancy, implying an interaction with an overexpressed HLA-E in the endothelial cell membrane (202). Endothelial HLA-E recognition by peripheral NK cells most likely modifies NK cell activity toward a low cytotoxic profile and promotes the production of pro-inflammatory cytokines such as TNF-α and IFN-γ (203, 204). A possible role of terminal α2-3 sialic acid in the HLA-E α1-domain could be relevant to explain this activation profile in peripheral NK cells mediated by NKG2 receptor activation in severe stages of preeclampsia. We wonder if changes in the endothelium and innate response cell (mainly NK cells) activation and cytokine influence the systemic inflammatory response observed during pregnancy. Although the putative role of cytokines produced by peripheral NK cells in the systemic inflammatory response of hypertensive disorders in pregnancy remains unknown, we propose that peripheral NK cells modulate their cytokine profile in preeclampsia *via* HLA-E signaling or lectin-like receptors such as members of NKG2 family, as previously reported (29).

There is little knowledge about glycan recognition in the context of NK cell activation. Previous studies identified glycophorin A, glycodelin-A, and sialic acid-related structures (sialyl Lewis X, among others) as negative modulators of NK cell-mediated cytolysis (205–207). Indirect evidence suggests that glycan recognition may activate NK cells during systemic inflammatory response in preeclampsia, including differential expression of lectin-like receptors or sialic acid-recognition receptors from the Siglec family (208, 209). Ibeto et al. (210) evaluated the glycosylation profile of hCG isolated in urine from women at 7 and 20 weeks of gestation (first and second trimesters, respectively) and urine samples from women suffering from gestational trophoblastic disease. Ibeto et al. (210) found mono-antennary, bi-antennary, tri-antennary, and tetra-antennary *N*-glycans in serum hCG from early and late gestation and significantly lower hCG containing bisected *N*-glycans in patients with the gestational trophoblastic disease. Ibeto et al. (210) suggested that hCG-related bisected type *N*-glycans may directly suppress NK cell cytotoxicity, consistent with the report of Chen et al. (43).

Campuzano et al. (211) investigated the effects of placental glycans on NK cell activity using a peripheral NK cell with a full-term human villous tissue co-culture system to assess whether changes in glycosylation profile could account for differences in cytokine production and cytotoxicity. Peripheral mononuclear leukocytes-containing NK cells co-cultured with fresh villous tissue or BeW0 cells showed that CD56Bright NK cells did significantly produce higher concentrations of IFN*-*γ and TGF-β than CD56Dim NK cells, consistent with a previous report in which circulating CD56dim and CD56bright NK cells exhibited predominantly cytotoxic and cytokine-producing phenotypes, respectively (211). Besides, blocking glycosylation in a BeWo cell syncytialization model using *N*-glycosylation inhibitors (e.g., castanomycin and tunicamycin) revealed differences in intracellular IFN-γ production by CD56Bright NK cells in co-culture (211). It demonstrated that co-culture of primary placental villous tissue with the choriocarcinoma cell line BeWo modulates NK cell function through specific glycan expression. Tunicamycin specifically inhibits N-linked glycosylation by preventing core oligosaccharide addition to the nascent protein, and thereby, it may interfere with specific ligand-receptor interaction by a decrease of the trophoblast protein synthesis(Surani, 1979). Additionally, castanospermine is a potent inhibitor of alpha- and beta-glucosidases and alters the distribution of insulin-like growth factor (IGF) receptors in villous tissue explants, which reduces the effect of IGF (212).

Based on this, it has been proposed that end-glycans of glycoproteins participate in the syncytiotrophoblast–NK cell interaction during pregnancy inflammatory response regulation (**Figures 3A**, **B**). Thus, increased expression of sialic acid binding immunoglobulin-like lectin-6, a unique sialic acid receptor in the placenta, was found during severe preeclampsia (213), and its concentrations were related to the severity of the pathology (214, 215). A case report of a woman presenting gestational diabetes mellitus, overweight, and severe PE, lower staining of Gal-GlcNAc (detected by the DSA lectin), mannose (detected by the GNA lectin), and Sia α2-3 (detected by the MAA lectin) was found in TfR. Accordingly, we propose that increased α2-3 sialyltransferase activity in this woman could explain the increased sialylation in TfR from placental villi (182).

The improved protein stability conferred by sialic acid may be advantageous because there is an increase in inflammatory status ROs production in preeclampsia (reviewed in 216–218). Protein stability is influenced by ROs (219). In placental malaria, increased chorionic villi α2,6 sialylation may influence intervillous placental parasite density as a compensatory response to *Plasmodium falciparum* infection (220). Preeclampsia and placental malaria are diseases with a placental and systemic inflammatory response in which NK cell activity increases progressively throughout gestation and may affect the communication between syncytiotrophoblast and immune cells (123, 221–223).

### 1.11 An emerging role of EVs in the physiology of the maternal-fetal interface and the exacerbated systemic inflammatory responses?

During the differentiation of cytotrophoblast and fusion to form the syncytiotrophoblast, glycosylation patterns change in a carbohydrate-driven pattern to provide the immunosuppressive microenvironment required for successful placentation, which appears impaired in pathological conditions of gestation. The released EVs from syncytiotrophoblast also carry various conjugated molecules in the maternofetal interface II context. Their size ranges from 30 to 150 nm, increasing in patients with an exacerbated systemic inflammatory response (224, 225). Studies have shown that levels of EVs increase in patients with systemic inflammatory response (224, 225), and this increase is even more pronounced in preeclampsia, where EVs have been found to increase three-fold compared to normal gestation (226, 227). To better understand the composition of EVs in placenta from normal and preeclamptic women, researchers have used perfusion systems to identify their main components (227). These EVs appear to play a role in the transfer of intercellular biomolecules such as flt1, the VEGF membrane receptor, as suggested by recent studies (228, 229). Thus, the exacerbated systemic inflammatory response in EP is associated with the number and variety of EVs found in healthy pregnancies (226, 227). The preeclampsia-associated chronic placental hypoxia and inflammatory status promote EVs shedding from syncytiotrophoblast to the maternal–fetal interface 2 and increase the systemic inflammatory response (230–233). Research suggests that glycans play a crucial role in EVs biogenesis, cellular recognition, and cell receptor interaction. In particular, mannosylated and sialylated N-glycans, with their antennae structures, are believed to mediate important biological functions such as inflammatory cascade activation and other paracrine events (234, 235) Thus, some glycan biomarkers, such as α-2-6 sialic acid and Gal or terminal GlcNAc in complex N-glycans, have been identified in syncytiotrophoblast-derived EVs (236). However, despite advancements in understanding the roles of syncytiotrophoblast-derived EVs glycans, their functional capabilities and interactions with target endothelial cells in both the glomerular filtration barrier and cerebral microcirculation remain largely unknown. Research conducted by our group has shown that serum from women with preeclampsia can affect the function of the glomerular filtration barrier in an *in vitro* model (237–239). This effect appears to be caused by a reduction in the anionic charge of the glomerular endothelium, allowing selective filtration of blood components into the urine. This raises the question of whether the glycans present in EVs from the placenta of women with preeclampsia could modify the permeability of the glomerular endothelium and cerebral microvasculature to albumin, and if they could affect endothelial integrity (43, 145, 240).

These EVs could explain the connection between the maternal–fetal interface and target organs such as the blood–brain barrier and the glomerular filtration barrier, where glycans are the most promising exchange molecules for research purposes. Further research is needed to fully decipher the complex mechanisms at play in these important physiological processes.

### 1.12 Perspectives on the role of glycans in gestation and preeclampsia: Connecting the network

Gérard Chaouat proposed nearly a decade ago that the specific immune system evolved to be adapted to vertebrate placentation (in the sense of subordination) as a critical feature of successful vertebrate reproduction (241). Decidual and circulating immune cells must undergo a series of molecular adaptations to avoid conceptus rejection and allow successful embryo and fetus development. The profile of glycan expression at the maternal–fetal interface, the phenotype and functional profile of innate immune cells present in the decidua, the presence of regulatory T cells (242, 243), and the production of soluble factors related to apoptosis, among others, may be responsible for such adaptation process (243–245).

The presence of immunomodulatory glycans in trophoblast is one fundamental mechanism supporting successful placental reproduction, along with the selective expression of “friendly” natural killer cells and low expression and activity of effector T cells capable of exerting cytotoxic activity. Most cellular and molecular mechanisms of the effector adaptive immune response are absent or regulated at the maternal–fetal interface. On the contrary, decidual NK cells are the primary immune cell population in up to 85% of decidua and are responsible for decidualization in early human pregnancy. Interestingly, one of the most abundant proteins produced by syncytiotrophoblast is β-hCG, a placental hormone sharing *N*-glycans coupled to asparagine residues that function as a potent immunomodulatory and angiogenic glycoprotein inducing decidual NK proliferation *via* mannose receptor activation (127, 246, 247). In healthy dynamics that support placental angiogenesis, decidualization, and establishing the maternal–fetal interface, uterine dendritic cells recruit decidual NK cells. Thus, the placenta of pregnant mice depleted of decidual NK cells shows a reduced expression of α2,3-sialylation and O-glycans, whereas the expression of branched *N*-glycans and Gal-1 increased. Ablation of decidual NK cells in the pre-implantation period could influence VEGF-mediated angiogenesis changes and depend on GAL-1 signaling. Experimental murine models of abnormal pregnancy show that changes in trophoblast glycosylation patterns precede poor pregnancy outcomes such as intrauterine growth restriction (248–250).

In conclusion, the fact that fetal and maternal cells exchange, at least in specific windows and compartments during the gestation period, adds to our understanding of the complex interplay between the maternal endocrine and immune systems as part of the maternal, placental, and fetal physiological environments and the maintenance of a balanced, functional immune response. Furthermore, evidence supports the theory of the involvement of the innate immune response in mediating critical processes required for early placental development and maintenance, such as decidual growth-supported placental development. In that context—and evolutionary highly conserved—glycans emerged as crucial innate immune cell regulators that support the maintenance of gestation. Because glycan expression profiles are essential modulators of adaptive and effector immune functions, critical advances in understanding successful human and mammalian gestation may provide a comprehensive understanding of normal and pathological pregnancy conditions (**Figures 3C**, **D**). We contributed to the discussion on the role of glycans in the pathophysiology of hypertensive disorders of gestation, as proposed by Gérard Chaouat in criticizing the pitfalls of the old immunotropic hypothesis. The main findings are summarized in the **Table 1**.

**Table 1:** Hypothetical and confirmed role of glycans in physiological and complicated pregnancy.

Organ	Source	Glycan	Condition	Function/pathogenesis	Reference
Placenta	Extravillous trophoblast	TF antigen	Normal pregnancy	Probably migration through the extracellular matrix for promoting invasion	(93, 94)
	Sera samples	hCG (N-glycan glycoprotein)	Normal pregnancy	Promotes trophoblast invasion by its immunomodulatory action	(146, 147)
	Syncytiotrophoblast	highly sialylated N-glycan structures	Normal pregnancy	Syncytiotrophoblast resistance to NK cell—and other cytolytic leukocytes—mediated cytolysis	(43, 135)
	Terminal Villi	Mannose, sialic acid, and β-galactose	Severe preeclampsia	Increased expression	(251)
	Stromal cells/Stroma	Glycodelin-A	Normal pregnancy	Biantennary N-glycans with the bisecting GlcNAc sequence and sialylated complex N-glycans	(143–145)
	Cytotrophoblast/ Villous trophoblast	Lewis*Y*	Preeclampsia Unexplained miscarriage	Angiogenesis-related glycan	(148, 149)
	Syncitialized BeWo cells	Reduced α(2-3) sialylation	Choriocarconoma BeW0 cells	Immunorregulation of peripheral NK cell cytokine production	(211)
	Chorionic Villi	Gal-1	HELLP syndrome	Defective trophoblast invasion	(144)
	Capillary Endothelium	Increased Mannose and β-galactose; decreased sialic acid	Severe preeclampsia	Placentation and angiogenesis	(251)
Decidua	Decidual stroma	Biantennary de-sialylated non-fucosylated N-glycans	Normal pregnancy	Reduced expression	(141)
	Decidual stroma	Gal-1	Upregulated in Preeclampsia	Defective trophoblast invasion	(144)
	Placental tissues (decidua and trophoblast)	Bisecting GlcNAc	Early-onset severe preeclampsia	Reduced expression	(172, 182)

This manuscript was edited and proofread before being sent to Gerard Chaouat for final review and approval. However, we did have the misfortune of his death. Therefore, this is our tribute to “Gégé” (Gérard C. Chaouat, 1944–2021).

## Author contributions

JB-S conceived and wrote the manuscript and designed the cited studies. AG-G performed cited studies and discussed their results. JM-E. participated in the critical review and reduction of the manuscript. JQ-C. participated in the critical review and reduction of the manuscript and study design. All authors contributed to the article and approved the submitted version.

## In Memoriam

In Memoriam: Gérard C. Chaouat (1944–2021).
